# Acute Pleural Effusion in Children1Read at the Buffalo Academy of Medicine, October 15, 1896.

**Published:** 1896-12

**Authors:** J. C. Clemesha

**Affiliations:** 382 West Ferry Street


### ACUTE PLEURAL EFFUSION IN CHILDREN.[1]

By J. C. CLEMESHA, M. D., L. R. C. P., Lond., M. R. C. S., Eng.

LITTLE is said on this subject in the ordinary text-books on medicine, it seldom forms the subject for a clinical lecture in the hospitals of our medical colleges, and I fear the average graduate is sometimes seriously nonplussed when a case of this kind is first brought to his notice for diagnosis and treatment.

Dr. West, the founder of the first children's hospital, remarks, in his work on Diseases of children : "There are no cases in which it has happened to me to have to screen the mistakes of others so often as in cases of pleurisy and empyema."

It is a more common disease in childhood than is generally supposed, yet its presence is often overlooked, or the existence of mischief in the chest referred to a wrong cause. In some cases the condition of the chest is overlooked, the trouble being referred to the abdomen or brain. And there are certain reasons why mistakes do occur. The subjective and objective symptoms of this disease are, to some extent, peculiar in early life and serve to obscure the real nature of the affection, unless one be on his guard.

1. Read at the Buffalo Academy of Medicine, October 1F, 1896.

There is also the great difficulty met with in making a sufficiently complete examination of the chest in early life, to enable one to accurately appreciate the physical signs existing, and thus a precise diagnosis is often difficult to arrive at.

I purpose, first, to dwell on the points in which pleurisy in children differs from the same affection in adults, and afterward to point out and differentiate the chief diseases with which pleurisy in children may be confounded. In ordinary cases of acute pleurisy with effusion in adults, the diagnosis is comparatively easy: the pain in the chest, catching the breath, with fever and cough, and increased frequency of respiration, the friction sound, some impairment of movement and deficiency of vesicular murmur in the affected side in the first stage, followed, as the effusion increases, by bronchial breathing broncophony, egophony and eventually ending in silence; absence of breath sounds, too, beginning at the lower part of the lung and extending upward as the effusion fills the pleural sac.

The percussion note, at first dull, and higher in pitch, ending in complete dulness or flatness with a sense of resistance and solidity to the stroke, the dulness changing with change of position, the absence of vocal fremitus, diminished motion and increased measurement of the affected side, with the effacement and perhaps bulging of the intercostal spaces and the displacement of the adjoining viscera, all combine to make a diagnosis with little risk of error. In children, however, many of these symptoms may be absent, or if present, from the difficulty of eliciting information from the little patient, are not discovered.

Pain in the side is more frequently absent in children than in adults, and when it exists we cannot usually obtain precise information from the patient as to its locality. In some cases the pain may be referred to the abdomen and vomiting and purging may be the principal symptoms at the onset. Cough is commonly present and gives rise to pain, but in some cases the cough may be slight and may remain absent throughout the whole course of the disease. Or, again, the cough may be of the most violent and convulsive character, rivaling in severity that of whooping-cough.

The predominance of nervous symptoms in children is marked in some cases and the disease is often ushered in with intense headaches and screaming, attended by vomiting and purging, symptoms which divert the attention from the chest. The temperature in pleurisy at the onset may reach as high as 105°, remaining only a few days and then gradually falling until absorption has taken place, when it becomes normal. If the effusion becomes purulent, the temperature will again rise and remit—100° in the morning, 104° at night, until the pus is evacuated. The pulse is very quick and hard at the commencement; in cases of great effusion, small and weak. The urine is scanty, of high specific gravity, dark in color during the height of the fever. As the pleuritic effusion is absorbed, it becomes more abundant, lighter in color and of a lower specific gravity. Even in mild cases, there is considerable loss of strength and pallor. In chronic cases, emaciation occurs to a marked degree. Vomiting, a rare symptom in adults, occurs in about half of the cases as an early symptom in children.

In secondary pleurisies, as after scarlet fever, the symptoms may be only an increase of those of the previous disease, especially as in these diseases there may have been increased rapidity of breathing and a few râles in the chest.

*Physical Signs.*—In regard to physical signs, the diagnosis is rendered more difficult from the fact that some of the signs, available in the adult, are less available or absent in a child, and thus to the inexperienced or careless, giving rise to error.

Vocal fremitus and vocal resonance are much more difficult to obtain in children, and information derived from these signs is not valuable. On inspection will be seen feeble expansion of the affected side and as the effusion increases widening and obliteration of the intercostal spaces. Displacement of the heart to the right or left will be found in cases of extensive effusion. The liver and spleen may also be pushed downward, but this is difficult of detection and need not be looked for with any certainty, in that the chest wall yields more easily in the child and the force of pressure is not expended to anything like the same extent as in adults, in dislocating the heart and forcing down the diaphragm and abdominal viscera. It is, however, a mistake to say that displacement of viscera never takes place in children.

In percussion we have most valuable diagnostic information, but unless the chest be full of fluid there is not absolute dulness, and we must not look for that flat sound that we find in pleurisy of the adult. The apex of the affected side will give a high pitched note and pleurisy at the base is the most common cause of hyper-resonance at the apex. Persistent dulness on percussion, even with fair respiratory murmur, should always excite suspicion of effusion into the pleural cavity and the aspirator should be used. Auscultation, as a means of diagnosis, cannot be relied upon and may give rise to false conclusions. The friction sound, if present, owing to the difficulties in auscultation, will seldom be heard. It is common to find in the pleurisy of adults complete silence when there is much effusion; while in children, on the contrary, I think the rule is that modified respiratory sounds are heard. In my limited experience tubular or bronchial breathing was found over the affected side. In fact, so constant is tubular or bronchial breathing in this disease that Allbutt says:

"Its existence in the child with broncophony should lead us to expect fluid." But we may not even have this sign, for in one case that I saw there was at times fair vesicular breathing.

Moreover, there may be breathing vesicular in quality, but deficient in quantity, while the respiratory murmur on the healthy side may be so exaggerated as to mislead the practitioner and cause him to treat the wrong lung. This happened in a case that came under my father's notice and the physician who made the mistake was not a young practitioner either. I mention this to emphasise the difficulties of diagnosis.

On account of the smaller dimensions of a child's chest and thus a thinner layer of fluid, there is often with the bronchial breathing increased vocal resonance, a fact which renders the diagnosis still more difficult. But as I have before stated, vocal resonance is difficult to obtain and as a sign cannot be relied upon.

*Diagnosis.*—It may be considered an axiom that in all doubtful cases of acute diseases in children a careful examination of the chest is very important. Without it errors will surely occur. Especially in pleurisy there is so much difficulty in discovering the exact condition of the lungs that a superficial examination of the chest will result in a mistaken diagnosis.

In my remarks on diagnosis I refer to purulent pleurisy only, for except there be heart displacement or position, dulness and absence of breath-sounds, I think the diagnosis must be doubtful. In all cases that I have seen and in those coming under my father's notice, which have been verified by the hypodermic needle, there has been purulent exudation. Perhaps the serous effusion is rapidly absorbed, therefore giving no cause for further exploration and probably set down as pneumonia.

The chief diseases with which acute pleurisy may be confounded are pneumonia, phthisis, acute hydrocephalus, typhoid fever, hydrothorax, pericarditis and abdominal affections. The first three are the commonest causes of error and to these only will I refer.

*Pneumonia.*—The onset of pleurisy very much resembles that of pneumonia, and indeed, when we remember that according to some authorities pneumonia, in a large proportion of cases, precedes purulent pleurisy, it is not surprising that errors in diagnosis are made.

Sutherland states, *Lancet*, 1894, that out of seventeen cases of double empyema, fourteen, or 67 per cent., were preceded by lobar pneumonia, one by broncho pneumonia, and only two were regarded as primary, and Adam states that out of thirty-two cases of unilateral empyema 71 per cent. were preceded by pneumonia. Hood in *Lancet* of August, 1894, in a clinical lecture, states :

If in what is apparently a pure uncomplicated attack of acute lobar pneumonia the critical stage is absent and resolution does not take place in reasonable time, it behooves us to examine the chest with the most conscientious care. Of all the secondary conditions leading to the absence of crisis none is more common than effusion, and if we are not fully alive to this complication, it is easy to drift complacently into error with regard to the true pathology of the case. The fever does not fall, the lung does not resolve, day after day it is watched and the dulness and tubular breathing remain. Here the attack has commenced with all the characteristic symptoms of acute pneumonia. There have been no symptoms denoting that with the subsidence of the pneumonia inflammation, there has been a gradual steady effusion poured out into the corresponding pleural cavity.

Goodhart speaks in a similar manner as follows :

In pure lobar pneumonia we may confidently expect the duration of the disease to be short. In purulent pleurisy, on the contrary, there is no limit, no tendency to recovery unless the pus be evacuated. The patient goes on emaciating and this fact alone imperatively demands the aspirator in order to clear up the doubtful diagnosis. Nor should we be satisfied with aspirating the base of the lung, as the pus may be encysted and in various parts of the chest.

Goodhart reports three cases in which fluid was only found below the clavicle. Dr. Squire reports a case of pyothorax limited to the upper lobe of the left lung, following a pneumonia, and also mentions in his paper having seen several cases of empyema following a pneumonia. In one of the cases coming before my notice the pus was encysted and the opening made in the subclavicular region on the left side. Dr. Addison, in his twenty-seventh aphorism, writes: "When serous effusion is very considerable, giving rise to unequivocal broncophony, tubular breathing, want of resonance and vocal vibration, physical examination has repeatedly led to a mistaken belief that these signs resulted from a pneumonia or other consolidation of the lung;" and I have no hesitation in affirming with equal confidence that similar mistakes may occur even if the effusion be purulent.

The onset of pleurisy sometimes resembles very much the onset of cerebral mischief, so much so that two of the cases I have seen had their heads shaved and blistered. In one of the cases there was stupor, in the other delirium. There may be vomiting, headache and screaming, and the cough may be slight or quite absent. Hurried breathing would be explained by the fever, and unless the chest be carefully examined an error in diagnosis will be made. However, the onset of pleurisy is usually too acute and probably has not been preceded by loss of flesh, capricious appetite and general symptoms of failing health. In these cases careful examination of the chest clears up the case.

Empyema may be wrongly diagnosticated as phthisis, in cases where the pus is retained in the pleural cavity for some time and symptoms of emaciation, hectic fever and cough and the like are most prominent. With regard to physical signs, phthisis in children often attacks the base of the lungs, in adults the apex. Where the onset simulates abdominal mischief, the child may have pain in the abdomen or over the liver, together with vomiting and purging. Careful examination of both chest and abdomen is needed to decide which is the affected region.

From what I have said I think it will be inferred that in these obscure cases of acute chest disease in children, the only positive means of diagnosis is the use of the aspirator, the ordinary hypodermic syringe answering the purpose very well, a large needle being used. As already stated, exploration may have to be made at different points; usually, however, this is not necessary, as the effusion is in the lower part of the pleural cavity.

*Terminations.*—Where the effusion is serous, pleurisy in children is not a serious disease and generally clears up rapidly. And here I should like to ask, Is serous pleurisy in children often met with, and how is the diagnosis made possible? Does serous pleurisy become purulent, or is the effusion purulent from the beginning?

Goodhart says: "But while allowing this to be possible, I

know little to support it and, indeed, clinical facts all seem to me to prove that it is quite rare, and in no case, to my knowledge, where aspiration has been employed was the fluid other than pus. Jacobi has found pus on the fourth day. Circumscribed collections of pus in the pleura are not so uncommon as might be supposed from the space devoted to the subject in works on diseases of children. The termination of an empyema, if the pleural sac be not drained, will probably be disastrous. According to some writers absorption may occur, but this, I think, is exceedingly rare. The pus may make its way through the chest walls or bronchial tubes and be coughed up, but rarely into the abdominal cavity. The most common clinical course of an empyema, unless the pus be evacuated, will resemble that of phthisis cough, emaciation, hectic diarrhea and death.

When, however, the pleural sac is opened and the pus drained off, the termination is in recovery in a large proportion of cases in from five to ten weeks after operative procedure, leaving the lung unimpaired and the child soon convalesces and becomes robust and hearty, even where the emaciation has been extreme and the disease protracted.

In speaking of treatment, my remarks apply only to empyema, for serous pleurisy, if diagnosticated, calls for no special treatment differing from that in the adult and the effusion is usually rapidly absorbed. The treatment should properly come under the surgical section, for the medical treatment of empyema requires but brief notice, the indications to be met with as they present themselves, and I cannot emphasise too strongly the utter uselessness of trusting to medical treatment alone in such cases.

The duty of the medical practitioner is to explore, when in doubt; this in ordinary private practice may not be so simple as it appears, for the parents invariably oppose anything like operative procedure in the early stage of the disease, and it is only when they see the patient going from bad to worse that consent is given. The physician, too, may not be positive enough at first, the existence of doubt as to diagnosis and the occasional favorable indications in the course of the disease combine to delay the all-essential operation.

In using the exploring needle I think it is well to be prepared to proceed at the same time with incision and drainage if pus be found; and in private practice it is perhaps better to give a little chloroform before proceeding to aspirate, as the child will be sure to scream and resist, and it may be needful to make a number of punctures before finding pus, especially if it be encysted. If pus be found, proceed at once to the radical operation of incision and drainage. The removal of a portion of a rib, or ribs, is rarely necessary in the case of children. The point of election for incision is the sixth or seventh intercostal space in the posterior axillary line. The incision through the skin need not be a long one, as some advise, and the operation is completed by pushing into the cavity a pair of dressing forceps and then opening the blades wide enough to make the opening sufficiently large to permit the introduction of the drainage-tube. The tube should be as large as can be got in, of soft rubber and fenestrated. I speak of the tube being soft, as in one case that came under my notice the tube used was somewhat rigid and caused troublesome cough and irritation. The tube may be retained in place by passing a strong silk thread through the portion external to the chest and tying it round the body, fixing it in several places with adhesive plaster. The tube, with shields, as depicted in Hare's system of therapeutics, should not be used, as it is impossible to keep the parts under the shield clean. The tube may be covered with borated cotton, or finely picked oakum, which will absorb the discharge.

The dressing should be changed twice a day at first and afterward from time to time as required. There seems to be no rule as to the length of time the tube should remain in. One writer advocates removal at the end of two weeks, even when the discharge is free, holding that after the pressure is removed absorption of the remainder of the pus will take place. From cases noted I am of opinion that from four to nine weeks would be nearer the average.

Aspiration may be practised in cases of infants and very delicate children, or when the pus is in small quantities, with good results. Bowditch, Barlow and Goodhart report good results in such cases.

The practice formerly advised of washing out the pleura with some antiseptic solution has now been abandoned as not only useless but fraught with danger, and it certainly may cause considerable pain and irritation. Should the pus, however, be foul-smelling, antiseptic injections might be used, but even when this is the case, it is not absolutely necessary, as the discharge will sometimes become sweet as the drainage goes on. The exuberant granulations which may spring up round the tube sometimes alarm the friends, as also the occasional eczematous condition of the skin, but need give no cause for alarm, as the condition disappears with removal of the cause.

382 West Ferry Street.

